# Intramuscular tetanus neurotoxin reverses muscle atrophy: a randomized controlled trial in dogs with spinal cord injury

**DOI:** 10.1002/jcsm.12836

**Published:** 2021-10-27

**Authors:** Anna Kutschenko, Anja Manig, Angelika Mönnich, Beatrice Bryl, Cécile‐Simone Alexander, Martin Deutschland, Stefan Hesse, David Liebetanz

**Affiliations:** ^1^ Department of Neurology University Medical Center Göttingen Germany; ^2^ Neurological Department Medical Park Berlin Humboldtmühle Berlin Germany; ^3^ TierReha Alexander Tierarztpraxis für Rehabilitation Berlin Germany; ^4^ Neurological Referral Veterinary Surgery Berlin Germany

**Keywords:** Spinal cord injury, Dogs, Paraplegia, Paraparesis, Tetanus neurotoxin, Muscle thickness

## Abstract

**Background:**

Motor symptoms of spinal cord injury (SCI) considerably impair quality of life and are associated with a high risk of secondary diseases. So far, no pharmacological treatment is available for these symptoms. Therefore, we conducted a randomized, double‐blinded, placebo‐controlled study in dogs with spontaneous SCI due to disc herniation to test whether a reduction of spinal inhibitory activity by intramuscular injections of tetanus neurotoxin (TeNT) alleviates motor symptoms such as muscle atrophy or gait function.

**Methods:**

To this end, 25 dogs were treated with injections of either TeNT or placebo into their paretic hindlimb muscles. Effects of TeNT on muscle thickness were assessed by ultrasound, while effects on gait function were measured using the modified functional scoring system in dogs.

**Results:**

Four weeks after the TeNT injections, muscle thickness of the gluteus medius muscle (before median 1.56 cm [inter‐quartile range {IQR} 1.34–1.71 cm] and after median 1.56 cm [IQR 1.37–1.85 cm], *P*‐value 0.0133) as well as of the rectus femoris muscle (before median 0.76 cm [IQR 0.60–0.98 cm] and after median 0.93 cm [IQR 0.65–1.05 cm], *P*‐value 0.0033) significantly increased in the TeNT group. However, there was no difference in gait function between the TeNT and placebo groups. The treatment was well tolerated by all dogs without any signs of generalized tetanus symptoms or any spreading of effects beyond the lumbar level of the injected hindlimbs.

**Conclusions:**

With regard to the beneficial effects on muscle thickness, intramuscular injections of TeNT represent the first pharmacological approach that focally reverses muscle atrophy in SCI. Moreover, the study data support the safety of this treatment when TeNT is used at low dose.

## Introduction

Paralysis after spinal cord injury (SCI) not only causes considerably reduced quality of life of the affected patients^1^ and their caregivers^2^ but also leads to expansive socio‐economic burdens.^3^ In addition, SCI is commonly followed by a marked muscle atrophy resulting from muscle inactivity due to loss of central input.^4^ Moreover, the risks of secondary impairments such as neuropathic pain, urinary tract infection, bowel dysfunction, respiratory impairment, autonomic dysreflexia, osteoporosis, bone fractures, and pressure sores are significantly increased in SCI patients. At least the last points are usually a consequence of immobility, reduced activity, and disuse.^5^, ^6^, ^7^

Although strong research efforts have been made to improve sensorimotor functions of SCI patients, therapeutic options for these patients are still very limited. Various attempts to alleviate SCI symptoms by local or systemic stem cell application have led to mixed results with little or no relevant clinical benefit so far.^8^, ^9^, ^10^ More encouraging therapeutic effects result from approaches that aim at locomotor restoration by activating spinal locomotor circuits that are deprived from cortical input in SCI. In this context, promising experimental results have been reported from a combination of electrical stimulation and intraspinal pharmacological treatment. However, the transfer of this technology into clinical application still faces serious technical challenges.^11^, ^12^, ^13^, ^14^

For this reason, we want to put forward a novel pharmacological approach to alleviate in SCI motor symptoms by reactivating spinal motor neurons using targeted injections of low‐dose tetanus neurotoxin (TeNT).

The idea of making use of this highly potent neurotoxin as a possible pharmacological treatment in SCI goes back to the Moldovan neurologist Boris Sharapov and to the time of World War II. Sharapov reported three gunshot wounded patients, two with paraplegia and one with hemiplegia, who all three accidentally also developed tetanus infections. In the further course tonic contractions, stiff movements as well as active movements arose in his patients. Within a few days, he stated that after all, the hemiplegic patient had been completely restored. Based on his observations of these coincidental tetanus infections, he reasoned that TeNT had positively stimulated the preserved neurons and postulated its potential use as a therapeutic agent.^15^

Tetanus neurotoxin is a 150 kDa protein produced by the anaerobic bacterium *Clostridium tetani* . After binding to the presynaptic membrane of the neuromuscular junction, TeNT is retroaxonally transported to the ventral horn. At the level of the spinal cord, it is then translocated to spinal inhibitory interneurons where TeNT blocks the release of the inhibitory neurotransmitters glycine and γ‐aminobutyric acid (GABA) by cleaving vesicle‐associated membrane protein (VAMP)/synaptobrevin that is a protein of the soluble N‐ethylmaleimide‐sensitive factor attachment protein receptor (SNARE) complex of the neurotransmitter containing synaptic vesicle^16^, ^17^ As a consequence, TeNT leads to a focal disinhibition of the lower motor neurons and thus to a therapeutically desired muscle tone elevation within the targeted muscles.^18^, ^19^, ^20^

In our aim to explore this therapeutic potential of TeNT, we recently demonstrated in a case series of four SCI dogs that low‐dose injections of TeNT into paretic muscles lead to a facilitation of lower motor neurons and thereby not only to a reverse of muscle atrophy but also to an improvement of stand and gait function.^20^

Here, we present at first a prospective, double‐blinded, randomized, and placebo‐controlled study to further investigate the potential of intramuscular injected low‐dose TeNT in 25 dogs suffering from SCI due to degenerative disc herniations. The chondrodystrophic canine breeds are above others very much at risk for such herniations and therefore seem to be very suitable for the investigation of therapeutic effects on SCI‐related paraplegia.^21^, ^22^, ^23^, ^24^, ^25^ However, one of the most important features of this naturally occurring SCI animal model results from the anatomical specialty of the canine spine, that is, having the spinal cord extending down to the sixth lumbar vertebra so that lumbar disc herniation in canines would lead to SCI resulting in cross‐sectional symptoms, in contrast to a radicular or cauda syndrome that would be developed in man after lumbar disc herniation.^25^

In our study, 25 dogs were randomized and intramuscularly injected with either TeNT or placebo into their hindlimbs. During a follow‐up visitation, effects on gait function and on muscle atrophy were assessed by a modified Olby gait score^23^ and by ultrasound, respectively.

Although no significant effect was seen in the functional gait score, our data reveal that intramuscularly injected TeNT is able to reverse muscle atrophy in SCI, a feature that has never been reported before for a drug.

## Materials and methods

### Study design and animals

This prospective, randomized, double‐blinded, placebo‐controlled single‐centre (University Medical Center Göttingen) clinical trial in dogs with SCI was conducted and reported according to the CONSORT guidelines. It was approved by the Animal Care Committee of the University Medical Center of Göttingen, Lower Saxony, Germany, and by the Niedersächsisches Landesamt für Verbraucherschutz und Lebensmittelsicherheit (LAVES), Braunschweig, Lower Saxony, Germany (AZ 16/2199).

The study population was restricted to dogs with paresis of the hindlimbs due to SCI. Dogs were recruited via a printed and online advertisement in a dog owner's journal with readership within German‐speaking countries. The study aimed to recruit at least 12 dogs per treatment arm.

Inclusion criteria were non‐acute paresis or paralysis of one or both hindlimbs due to lumbar SCI with at least 6 month time between the onset of symptoms and study initiation. Dogs with other causes of paresis or paralysis, for example, radiculopathies, were excluded. To this end, we performed needle electromyography of the hindlimbs of each screened dog (gluteus medius, quadriceps femoris, gastrocnemius, and tibialis cranialis muscles). Further exclusion criteria were intolerance of daily handling and co‐morbidities that might affect recovery of neurological function. After screening, 25 dogs in total entered the study. A detailed list of animal data including disease history is provided in *Table* 1.

**Table 1:** Animal data

Subject #	Age (years)	Weight (kg)	Race	Height of injury	SCI to baseline visit (months)	Operation to baseline visit (months)	TeNT/placebo	Total dosage TeNT (pg)	MRC left	MRC right
1	14	11	Shih Tzu	L1–L2	36	36	Placebo		3	<3
2	5	17	French Bulldog	L1–L3	22	21	Placebo		<3	<3
3	5	13	English Cocker Spaniel	T12–L3	15	15	Placebo		<3	<3
4	4	10	French Bulldog	L1–L2	9	9	TeNT	1875	4	4
5	5	8	Mixed breed	L1–L2	n/a	n/a	TeNT	2063	3	3
6	8	10	Dachshund	T13–L1 and L2–L4	16	15	TeNT	4113	<3	<3
7	8	25	Mixed breed	L1–L3	17	17	TeNT	4313	4	4
8	10	8	Prason Russell Terrier	T11–T13	19	19	Placebo		<3	<3
9	11	22	Airedale Terrier	T11–T12	13	13	Placebo		<3	<3
10	4	36	Golden Retriever	T10–T11	44	n/a	TeNT	9975	<3	<3
11	8	10	Mixed breed	T9	96	96	TeNT	2734	3	4
12	7	4	Mixed breed	L1–L2	68	68	Placebo		3	3
13	10	41	Mixed breed	L3	23	n/a	TeNT	9750	4	4
14	3	21	Mixed breed	T12–L1	n/a	n/a	Placebo		3	4
15	8	40	Leonberger	L3–L4	18	n/a	Placebo		5	<3
16	8	7	Dachshund	T11–T12	56	56	Placebo		<3	3
17	2	8	French Bulldog	T13–L1	16	16	TeNT	2035	4	4
18	7	6	Pekingese	T10	48	n/a	TeNT	2700	<3	<3
19	9	8	Mixed breed	T11–T12	52	51	Placebo		<3	<3
20	3	8	Mixed breed	T8–T10	37	n/a	Placebo		<3	<3
21	9	8	Mixed breed	L1–L2	16	16	TeNT	4113	<3	<3
22	10	12	Boston Terrier	Unknown	12	n/a	TeNT	2100	3	3
23	4	12	Mixed breed	T12–L1	48	44	Placebo		4	4
24	5	8	Mixed breed	L2	30	30	TeNT	3638	<3	<3
25	5	20	Mixed breed	T11–T12	9	n/a	TeNT	7000	<3	<3

Comparison of demographic and clinical history data of participating dogs with SCI. The dogs were randomly divided into two groups and were injected with either TeNT or placebo. The individual total dosage of TeNT (pg) is stated for each TeNT‐injected dog. Medical Research Council scale (MRC) median of medial and lateral gastrocnemius, medial and lateral vastus, gluteus medius, rectus femoris, and tibialis cranialis muscles is given for each side.

The dog owners were informed of the clinical trial and had to sign an informed consent for each dog regarding the compassionate use of low‐dose intramuscular injections of TeNT. In addition, a written informed consent for publication was obtained.

Dogs were randomized prior to the treatment by the person preparing the injections that was not involved in data collection or outcome assessment. Because the weight range of the dogs was very wide (4.3–40 kg), five weight groups were created (Group 1: 30–41 kg; Group 2: 17–25 kg; Group 3: 10–16 kg; Group 4: 7–9 kg; and Group 5: 4–6 kg) to equally distribute both treatment arms (TeNT vs. placebo) in a 1:1 ratio in each weight group (*Table* 2). Neither the person performing the injections nor the persons obtaining the data nor the dog owners were aware of the treatment condition. Unblinding followed after all data including the assessment of gait via the external examiner were obtained. Because of logistical limitations, no long‐term data were obtained.

**Table 2:** Weight‐adapted randomization of the dogs

Group	Weight range (kg)	Subject	Weight (kg)	TeNT/placebo
1	30–41	#10	36	TeNT
		#13	41	TeNT
		#15	40	Placebo
2	17–25	#2	17	Placebo
		#7	25	TeNT
		#9	22	Placebo
		#14	21	Placebo
		#25	20	TeNT
3	10–16	#1	11	Placebo
		#3	13	Placebo
		#4	10	TeNT
		#6	10	TeNT
		#11	10	TeNT
		#22	12	TeNT
		#23	12	Placebo
4	7–9	#5	8	TeNT
		#8	8	Placebo
		#16	7	Placebo
		#17	8	TeNT
		#19	8	Placebo
		#20	8	Placebo
		#21	8	TeNT
		#24	8	TeNT
5	4–6	#12	4	Placebo
		#18	6	TeNT

The dogs were grouped into five weight groups. Treatment condition was randomized within these weight groups.

### Processing of tetanus neurotoxin

Tetanus neurotoxin was provided by courtesy of Dr Andreas Rummel (Institute of Toxicology, Hannover Medical School, Hanover, Germany). Aliquots of 5 ng TeNT/mL (stored at −20°C) were thawed at room temperature and diluted in phosphate‐buffered saline with 0.1% bovine serum albumin to a final concentration of 625 pg TeNT/mL. This was performed directly before injection in order to reduce the risk of instability of the toxin. The placebo consisted of phosphate‐buffered saline with 0.1% bovine serum albumin.

### Intramuscular injection

The dosage of TeNT to be injected was calculated based on our previous *in vivo* studies in mice and dogs.^19^, ^20^ Using an automated running wheel paradigm, TeNT‐induced focal increase in muscle tone of the injected murine hindlimbs could be quantified.^19^ Based on the ratio between weight of dogs and weight of mice, the individual weight‐adapted dosage of TeNT was calculated for each dog. Before injection, the dogs were assessed for severity of the paralysis of each hindlimb and appropriate muscles were chosen.^20^ The functional state of each individual muscle was measured by Medical Research Council (MRC) scale (*Table* 1). Depending on MRC scale, different volumes of the diluted weight‐adapted TeNT respectively placebo were injected (100 % in case of MRC < 3, 75 % in case of MRC = 3, 50 % in case of MRC =4)      (*Table* 3). Using a 30 G needle, volumes between 200 and 1300 μL per muscle were injected, which in the case of the TeNT‐injected dogs corresponds to a dose of 125–812.5 pg TeNT. Injections were performed as a single bolus to each muscle; 24 of the 25 dogs received bilateral injections. One dog (dog #15) received injections limited to the right hindlimb because the left limb was not affected. The muscles injected in each hindlimb were the medial and lateral gastrocnemius (47/49 hindlimbs), the medial and lateral vastus (49/49 hindlimbs), the gluteus medius (47/49 hindlimbs), the rectus femoris (46/49 hindlimbs), and the tibialis cranialis muscle (28/49 hindlimbs).

**Table 3:** Calculation of injected volume

Weight (kg)	Dosage 100% (μL)	Dosage 75% (μL)	Dosage 50% (μL)
4	272	204	136
5	315	236	158
6	359	269	179
7	391	293	196
8	435	326	217
9	467	351	234
10	500	375	250
11	533	399	266
12	565	424	283
13	598	448	299
14	630	473	315
15	652	489	326
16	685	514	342
17	717	538	359
18	750	563	375
19	772	579	386
20	804	603	402
21	826	620	413
22	848	636	424
23	880	660	440
24	902	677	451
25	924	693	462
26	957	717	478
27	978	734	489
28	1000	750	500
29	1022	766	511
30	1043	783	522
31	1076	807	538
32	1098	823	549
33	1120	840	560
34	1141	856	571
35	1163	872	582
36	1185	889	592
37	1207	905	603
38	1228	921	614
39	1250	938	625
40	1272	954	636
41	1293	970	647

TeNT dosage was calculated in relation to the dogs' weight. This individual TeNT dosage was then assigned to the corresponding volume of the stock solution (625 pg TeNT/mL) in order to maintain blinding of the injecting person. The dogs were injected according to the severity of their paralysis as assessed by the Medical Research Council (MRC) scale. They received 100% (MRC scale <3), 75% (MRC scale = 3), or 50% (MRC scale = 4) of the weight‐adapted volume.

### Evaluation of muscle thickness using ultrasound

To assess effects of TeNT on muscle thickness, we measured the thickness of the rectus femoris and the gluteus medius muscle via ultrasound (Esaote, MyLab; 10 MHz) before and 4 weeks after the injections. For the rectus femoris muscle, we positioned the ultrasonic probe in the middle of an assumed line between the patella and hip,^20^ and for the gluteus medius muscle, we positioned the ultrasonic probe in the middle between the iliac crest of the pelvis and the greater trochanter of the femur.

### Gait assessment

Each dog was examined before and 4 weeks after the injections. Nociception was evaluated via reaction to pain by pressuring the dog's nail bed of the claw. The gait of each dog was videotaped on a non‐slippery surface. The videotapes were cut into 30 s of slow motion covering the same amounts of steps from both sides and from behind.

We evaluated gait applying the functional scoring system in dogs (FSSD),^23^ which was modified for separate assessment of each hindlimb (mFSSD).^19^ The scale of the mFSSD ranks from 0 (plegia of hindlimb and no deep pain sensation) to 14 (normal pelvic limb gait). The higher the score, the better is the gait function.^23^ The videotapes were rated in non‐consecutive order by an experienced veterinarian and animal physiotherapist familiar with the mFSSD, who was not involved in the procedures of the study and blinded for the treatment condition but with knowledge of nociception to score accordingly.

### Evaluation of adverse events following injection of tetanus neurotoxin

Four weeks after the injections, we asked the dog owners for any effects beyond the regions of intramuscular injection. In addition, we clinically examined whether the dogs exhibited motor symptoms above the level of lumbar spine such as of the fore limbs, of the abdomen, or of further cranially paraspinal muscles. In addition, we investigated whether the dogs had painful muscle spasms at rest and/or during movement and whether the nociception was present. Moreover, the dogs were examined and the dog owners were asked for any signs of general symptoms such as dysphagia, dyspnoea, or fever. Also, the dog owners were asked for a change of stool and/or of urine control.

### Statistical analysis

To compare subject data of the placebo and TeNT groups, we showed the data as mean ± standard deviation as well as minimum and maximum, and an unpaired *t*‐test was performed.

To evaluate the effects on muscle thickness, non‐normalized data before and after the injections were compared for each individual muscle. To test for significant group differences, a Wilcoxon matched‐pairs test was performed. Furthermore, relative change in muscle thickness before and 4 weeks after injection was calculated as delta values for the placebo and TeNT groups, respectively.

In order to evaluate the effect on the mFSSD, the initial value was subtracted from the follow‐up value in order to obtain the change as a numerical value. Mann–Whitney *U* test was performed to test for significant differences in between the treatment groups. A *P* < 0.05* was set as significant, and a *P* < 0.01** was defined as highly significant. The statistics and figures were performed with GraphPad Prism 5 2007 for Windows (Version 5.01) and Microsoft Excel 2017 for Mac (Version 15.33).

## Results

### Study population

**Figure 1:**
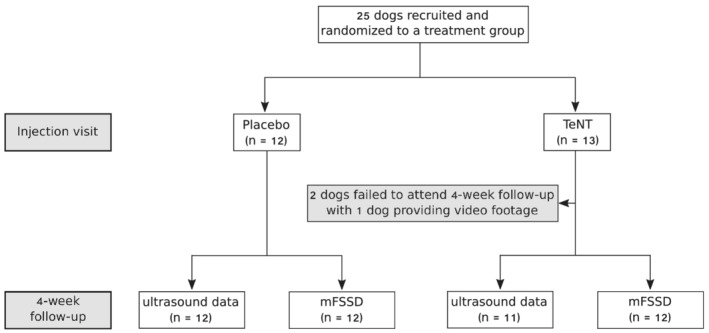
CONSORT flow chart. Study design and number of dogs recruited to each treatment group are documented.

Twenty‐five dogs were recruited and randomized into one of the two treatment groups (*Figure* 1). One dog (#17) was withdrawn at the time of follow‐up, in that the dog owners did not show up with the dog for this visit. One dog (#18) was not able to attend the follow‐up because of lack of transport means. But the dog owners of dog #18 provided video footage via mail for follow‐up. Thus, ultrasound data of 23 dogs (11 dogs randomized to the TeNT group and 12 dogs randomized to the placebo group) and gait analysis data of 24 dogs (12 dogs randomized to each treatment group) were available for evaluation.

The average age of the included 25 dogs was 7 years as the study groups were of mixed dog breeds, the average weight of the dogs was 14.9 kg (average weight placebo 14.3 ± 9.9 kg [minimum 4 kg and maximum 40 kg] and average weight TeNT 15.5 ± 11.5 kg [minimum 6 kg and maximum 41 kg], unpaired *t*‐test *P*‐value 0.77). All dogs had a history of SCI ranging from level T8 to L4 with an average of 31 months prior to injection (average duration SCI to injection placebo 34.9 ± 19 months [minimum 13 months and maximum 68 months] and average duration SCI to injection TeNT 28 ± 24.9 months [minimum 9 months and maximum 96 months], unpaired *t*‐test *P*‐value 0.47), resulting in various degrees of paresis and atrophy of the hindlimbs as well as heightened reflexes; 16 of the 25 dogs had undergone surgery mostly in form of decompressive operations with an average of 32 months (average duration operation to injection placebo 35.9 ± 19 months [minimum 13 months and maximum 68 months] and average duration operation to injection TeNT 28.4 ± 30.5 months [minimum 9 months and maximum 96 months], unpaired *t*‐test *P*‐value 0.56) prior to the injections (*Table* 1).

### Adverse events after intramuscular tetanus neurotoxin injections

None of the dogs had side effects concerning symptoms as occurring in an infection with *Clostridium tetani* such as generalized muscle spasms, dysphagia, dyspnoea, or fever. Furthermore, no cranial spread of muscle tone elevations beyond the lumbar level of the injected hindlimbs was observed.

### Dog owners reported effects of intramuscular tetanus neurotoxin injections

The owner of dog #22, treated with TeNT, reported an increased muscle tone of the hindlimbs that had a negative functional impact. However, this could not be objectified in the survey of the mFSSD in the 4 week follow‐up, which showed an improvement of 1 point. In a further seven out of the 12 dogs treated with TeNT (subjects #5, #6, #11, #13, #21, #24, and #25), the dog owners reported an increased muscle tone of the hindlimbs and in three out of these dogs (subjects #5, #21, and #24) about an improved control of urine and stool. Three dog owners from the 12 treated dogs with placebo (subjects #1, #14, and #16) reported the ability of the dog to walk a few more steps than usual.

Thirteen dog owners (nine from the placebo group and four from the TeNT group) did not find any changes in their dog.

### Effect of intramuscular tetanus neurotoxin injections on ultrasound‐assessed muscle thickness

**Figure 2:**
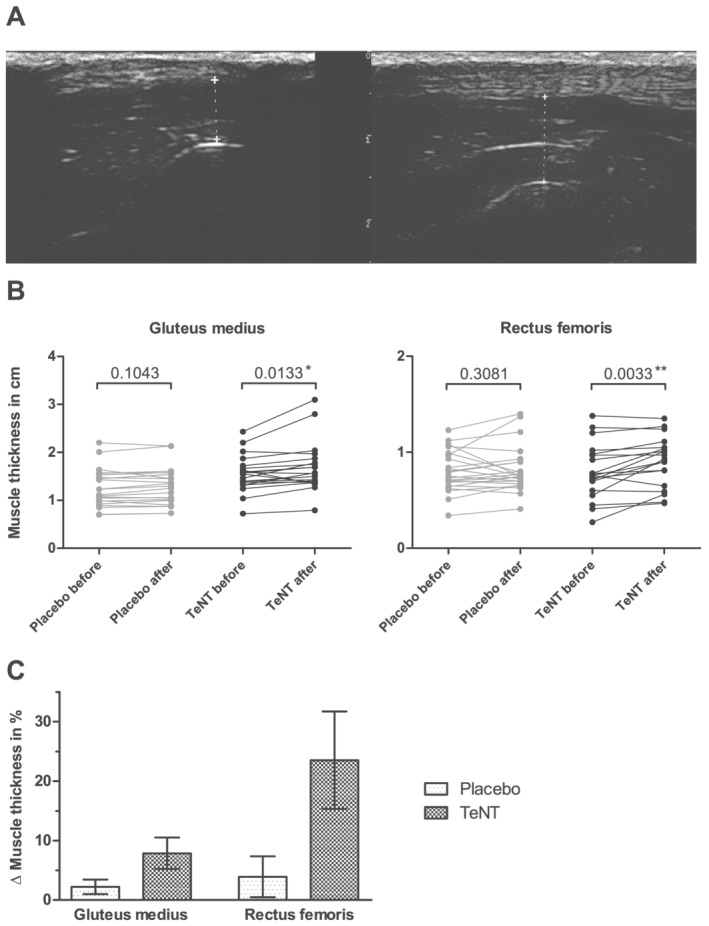
Ultrasound measurement of muscle thickness before and after TeNT injections. (**A**) Exemplary ultrasound image (ultrasound machine Esaote, MyLab, 10 MHz) of the rectus femoris muscle of dog #25 before (7.1 mm) and 4 weeks after (10.1 mm) TeNT injection. (**B**) Effect of TeNT injections on the thickness of rectus femoris and gluteus medius muscles assessed by ultrasound measurements as compared to placebo injections. Original values of muscle thickness before and 4 weeks after injection are shown for each individual muscle (rectus femoris muscle: placebo n = 23, TeNT n = 19; gluteus medius muscle: placebo n = 23, TeNT n = 20). Data are represented as original values (cm); p‐values (Wilcoxon matched pairs test) are stated above the brackets of the dot blot. The asterisk indicates significant results (p < 0.05*) and the two asterisks represent highly significant results (p < 0.01**). (**C**) Relative change of muscle thickness of gluteus medius muscle and rectus femoris muscle before injection and 4 weeks after the injection was calculated as delta values in per cent (rectus femoris muscle: placebo n = 23, TeNT n = 19; gluteus medius muscle: placebo n = 23, TeNT n = 20). Data are represented as mean with standard error of the mean. Neither the investigator nor the dog owner were aware of the treatment condition.

We obtained ultrasound data from 23 dogs as dog #17 and dog #18 did not attend the 4 week follow‐up. If either the gluteus medius or the rectus femoris muscle was not part of the injection scheme, ultrasound of that muscle was not performed. This resulted in total measurements of 43 gluteus medius muscles and 42 rectus femoris muscles. In the TeNT group, the muscle thickness significantly increased 4 weeks after the injections compared with the individual baseline muscle thickness before the injections (gluteus medius muscle: before median 1.56 cm [inter‐quartile range {IQR} 1.34–1.71 cm] and after median 1.56 cm [IQR 1.37–1.85 cm], Wilcoxon matched‐pairs test, a *Figure* 2). In the placebo group, the muscle thickness did not significantly increase 4 weeks after the injections compared with muscle thickness before injections (gluteus medius muscle: before median 1.11 cm [IQR 0.92–1.53 cm] and after median 1.25 cm [IQR 1–1.54 cm], Wilcoxon matched‐pairs test, *P*‐value  0.1043; rectus femoris muscle: before median 0.75 cm [IQR 0.65–0.97 cm] and after median 0.75 cm [IQR 0.67–0.90 cm], Wilcoxon matched‐pairs test, *P*‐value  0.3081; *Figu* *re* 2B).

Relative change of muscle thickness before and 4 weeks after injection was calculated in per cent as delta values (gluteus medius muscle: placebo mean 2.2% ± standard error of the mean [SEM] 1.2% and TeNT mean 7.9% ± SEM 2.6%; rectus femoris muscle: placebo mean 3.9% ± SEM 3.4% and TeNT mean 23.5% ± SEM 8.2%; *Figure* 2C).

### Effect of intramuscular tetanus neurotoxin injections on gait performance

**Figure 3:**
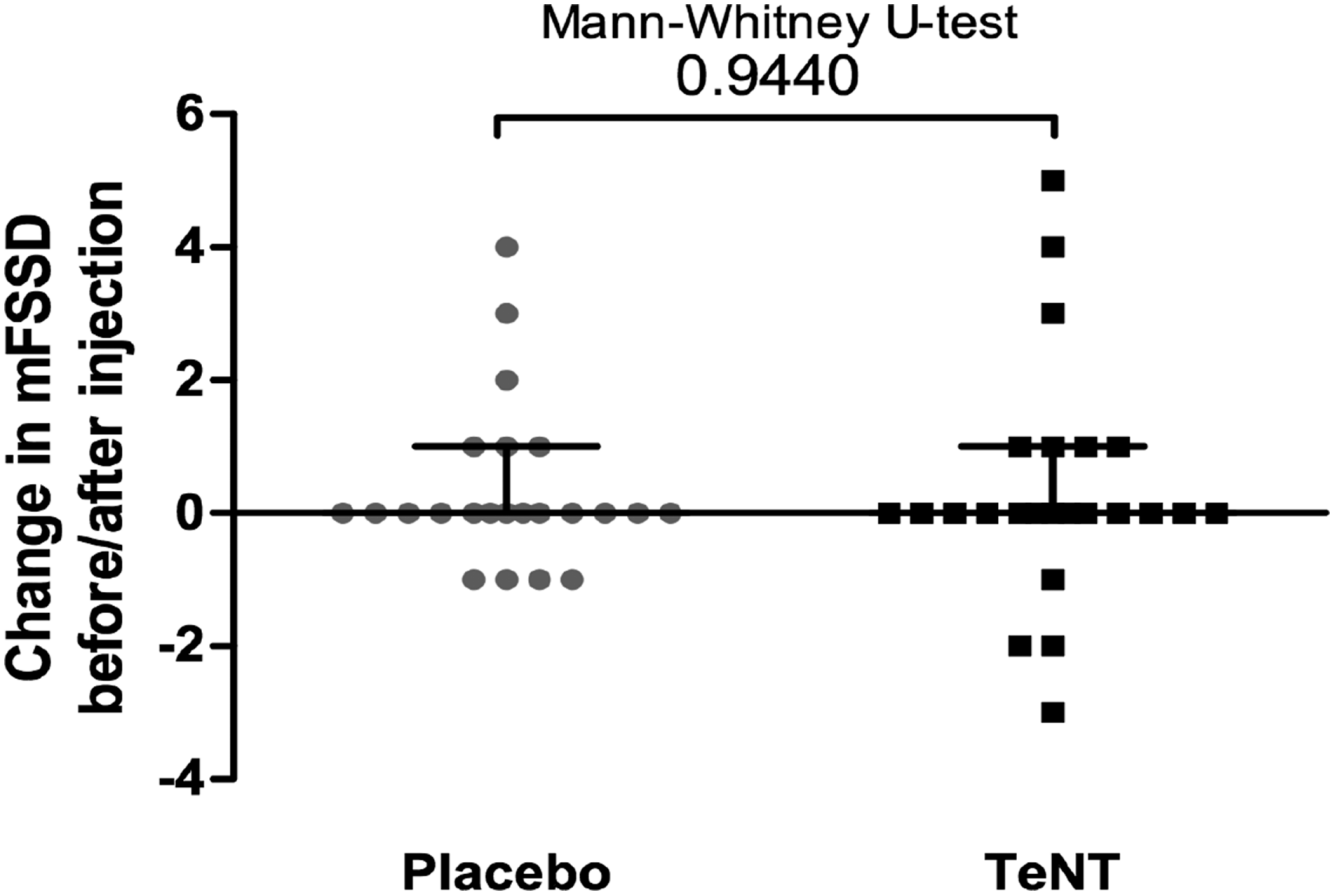
Effect of TeNT injection on gait performance. The change in the modified functional scoring system in dogs (mFSSD) before and after the injections is stated for both sides individually. A higher score indicates an improvement of gait function. Mann–Whitney *U* test was performed to test for significant differences between placebo‐injected hindlimbs (*n* = 23) and TeNT‐injected hindlimbs (*n* = 24). The *P*‐value is stated above the bracket of the dot plot diagram.

We obtained video footage of 24 dogs with subject #18 sending video footage via mail for follow‐up. Dog #15 was only injected at the affected right side so that the mFSSD was therefore rated only at this side. Altogether, 24 hindlimbs were rated in the TeNT‐injected group and 23 hindlimbs in the placebo‐injected group (*Table* 4). Nociception did not change after injection. No effect on mFSSD was seen in 13 hindlimbs of each treatment group. An improvement of gait, assessed by a positive value of change in the mFSSD, could be observed in seven hindlimbs of the TeNT group (29.2%) and in six hindlimbs of the placebo group (26.1%). A worsening of gait function, measured by a negative value of change in the mFSSD, was present in four hindlimbs in each treatment group; however, the worsening was more pronounced in the dogs of the TeNT group (dogs #4, #5, and #18). Accordingly, no difference between placebo‐injected and TeNT‐injected groups was found in the Mann–Whitney *U* test with regard to the effect on mFSSD (median placebo 0 [IQR 0–1] and median TeNT 0 [IQR 0–1], Mann–Whitney *U* test, *P*‐value  0.944, *Figure* 3).

**Table 4:** Effect of TeNT injections on gait performance as assessed by the modified functional scoring system in dogs (mFSSD) before and 4 weeks after the injections of TeNT or placebo

	TeNT							Placebo					
Subject #	mFSSD						Subject #	mFSSD					
	Right			Left				Right			Left		
	Before	After	Change	Before	After	Change		Before	After	Change	Before	After	Change
4	7	5	−2	5	6	1	1	5	5	0	6	10	4
5	0	0	0	3	0	−3	2	0	0	0	0	0	0
6	0	0	0	0	0	0	3	0	3	3	4	4	0
7	9	9	0	10	11	1	8	0	0	0	0	0	0
10	7	7	0	7	7	0	9	0	0	0	0	0	0
11	10	13	3	12	12	0	12	9	10	1	9	11	2
13	5	5	0	0	0	0	14	7	7	0	6	6	0
18	5	5	0	6	4	−2	15	3	3	0			
21	3	2	−1	3	3	0	16	0	0	0	0	0	0
22	12	12	0	12	13	1	19	6	5	−1	4	3	−1
24	4	4	0	0	4	4	20	6	7	1	7	6	−1
25	0	5	5	4	5	1	23	8	9	1	7	6	−1

The scores of each dog are stated from both sides individually. Because dog #15 was only injected at the affected right side, mFSSD is available from this side only. A higher value indicates a gait recovery. Likewise, a positive value of change in mFSSD specifies an improvement of gait performance.

From individual video footage of dog #5, the worsening of gait performance could be attributed to a more pronounced increase in muscle tone of the hindlimbs preventing the left hindlimb from performing minimal non‐weight‐bearing protraction of the pelvic limb that was still possible before injection of TeNT. It is noticeable with this dog that free standing was possible for several seconds after the injections but with collapsing of the hindlimbs when starting stepping with the front limbs. According to individual video footage of dog #18, this dog also clinically showed an increased muscle tone of the hind legs, which allowed only a non‐weight‐bearing protraction instead of the previously still possible weight‐bearing protraction.

## Discussion

Spinal interneurons that physiologically inhibit spinal motor circuits are a potential target to regain motor function by decreasing spinal inhibition in diseases of the central nervous system like SCI, multiple sclerosis, or stroke.^12^, ^14^, ^15^, ^20^ This double‐blinded, randomized, and placebo‐controlled clinical trial investigated the efficacy of intramuscular injections of low‐dose TeNT for the treatment of motor symptoms in SCI. In our proof‐of‐concept study, we specifically looked at improvements in gait function as well as on muscle atrophy. Because TeNT is one of the most neurotoxic bacterial proteins known, its safety has been thoroughly monitored.

Although a consistent effect on gait function of the TeNT‐treated dogs was not detectable at the 4 week follow‐up, the ultrasound‐based data on muscle size revealed that TeNT injections into paretic muscles of SCI dogs are effective to focally reverse SCI‐related muscle atrophy within 4 weeks. Despite the high toxicity of the protein, low‐dose TeNT turns out to be safe and well tolerated by all participating dogs.

Studies using animals with spontaneously occurring diseases are a promising approach for the translation of new therapies into human use.^24^ In this regard, especially chondrodystrophic dog breeds, which in up to 20% develop SCI due to spontaneous thoracolumbar disc herniation,^23^, ^25^ are considered as favourable animal model of SCI, because it is naturally occurring and also represents a common case in veterinary practice. SCI in these dogs comes along with a clinical presentation, pathophysiology, histopathological findings, and outcomes that are comparable with SCI in humans. Its overall better reflection of clinical reality as compared with classic experimental animal models makes this canine model highly suitable for translational studies on SCI.^24^, ^25^, ^26^

In the present study, low‐dose TeNT injections failed to induce a significant improvement of gait impairment in the participating SCI dogs. This is somewhat in contrast to our previously reported case series, where four treated SCI dogs had improved stance and/or gait function after TeNT injections.^20^ A major difference to our foregoing case series was the provision of regular physiotherapeutic treatment, which was not provided during the present study. The influence of this factor may have been underestimated in the current study and may represent a critical supportive element for turning the effects of TeNT into functional beneficial effects. This assumption is supported by the observation that despite the absence of mFSSD improvement, the dogs of the treatment group experienced an increase in muscle thickness, indicating that at least a muscular effect was present.

By the blockade of the release of inhibitory neurotransmitters at spinal interneurons, TeNT leads to a disinhibition of lower motor neurons. Increased activity of the lower motor neurons in turn results in more frequent contractions of the corresponding muscles fibres, that is, motor units. Finally, these mechanisms induce an increase in true muscle mass similar to the effect of muscle training. However, in the present study, this myofibrillar build‐up did not lead to an improvement in gait function. Most likely the missing effect on gait function is explained by an insufficient coordination of lower motor neuron activity of the affected segments. Without a minimum of coordination, muscle mass is gained but without function. This would explain the positive functional effects of regular physiotherapy in our previous study.^20^ In the context of TeNT‐induced spinal disinhibition, physiotherapy with weight‐supported gait and stand exercises could lead to an improved coordination of the lower motor neurons, which would be a prerequisite for beneficial effects on gait function.

Because in the present study injections lead to rather mixed results with regard to individual effects on gait function in the treatment group, which also is reflected by a larger dispersion of the mFSSD values when compared with placebo group, it is unlikely that possibly too small group numbers prevented the detection of a positive TeNT effect on gait. Rather, there are indications that the individual dosage of TeNT or the individual injection scheme may not have been suitable for improving the individual gait impairments. For example, for some dogs, the dosage has possibly been chosen too high, so that their increased muscle tone may have prevented a functional improvement (dogs #5 and #18). On the other hand, a possibly too low dosage may have been set for some other dogs, which gait score did not change at all (dogs #6, #10, and #13). A further interesting observation was that dogs that deteriorated in mFSSD during the study were already initially severely affected because of SCI; that is, only few steps (dog #5) or even no walking or standing (dogs #18 and #21) was possible. We noticed that some dogs, which were still able to walk more than 10 steps, benefited functionally from TeNT injection (dogs # 7, #11, and #22). Therefore, inappropriate dosage or the severity of the paralysis may have reduced the effectiveness of the treatment. These factors could at least partly explain the wide range of mFSSD values in the treatment group.

In contrast to the lack of beneficial effects on gait, TeNT injections resulted in a significant increase of ultrasound‐assessed muscle thickness as compared with placebo injections. These effects were present in both muscles examined, that is, the rectus femoris and the gluteus medius muscles, at the follow‐up 4 weeks after the treatment. This confirms the data from our previous case series on four dogs.^20^ Positive outcome on muscular trophics tended to be more pronounced in those dogs with a shorter interval between the SCI event and the injection than dogs with longer intervals. But nevertheless, even several years after SCI, TeNT treatment achieved a positive effect on muscle thickness. Such a positive and local effect of a drug on muscle mass has not yet been described for any other drug.

So far, only physical exercise and electric muscle stimulation are successfully applied to reduce muscle atrophy as indispensable components of the rehabilitation armamentarium.^27^, ^28^, ^29^ However, physical exercise requires high level of repetition and a sufficient level of voluntary muscle control to be beneficially effective. Drawbacks of electrical muscle stimulation are likewise the demand of high repetition rates and, in addition, its limitation to superficial muscle groups as well as the discomfort that is associated with the electrical stimulation. Therefore, an injectable drug would be a most valuable game changer in the treatment of muscle atrophy. Especially in SCI, profound muscle atrophy is often followed by bone fractures and pressure sores, which in turn lead to hospitalization and surgery and reduced quality of life of patients suffering from SCI.^5^, ^6^, ^7^

In addition to the positive effects on the dogs' muscle trophism, an improvement in control of bladder and bowels was reported by some of the dog owners. While this notion has only anecdotal character, as we did not evaluate or assess these functions systematically, it would be at least theoretically conceivable that the TeNT‐induced removal of spinal inhibition may have a positive effect on these functions, too. In this regard, for example, spinal electric stimulation has been reported to improve bladder control.^30^, ^31^ Future studies would have to address this potential of TeNT more specifically.

Safety of intramuscular TeNT injections is becoming a critical issue, when transferred to clinical application as TeNT is one of the most toxic proteins.^32^ In the present study, none of the 24 treated dogs showed any generalized symptoms or a spreading of TeNT effects on muscle areas above the injected level. Moreover, none of the participating dogs were reported suffering from severe or painful muscle spasms. With respect to negative functional effects of the TeNT treatment, one TeNT‐injected dog (dog #22) was reported by its owner to reveal a negative functional impact following an increased muscle tone. This disadvantageous effect was not reflected by a decline in the mFSSD. In contrast, four TeNT‐treated dogs worsened in mFSSD (dogs #4, #5, #18, and #21), in two of which an increased muscle tone of the hindlimbs caused a minor worsening of gait function.

However, in any case, this increased muscle tone was confined to the level of the injected muscles, which is in line with results of our previous studies on dogs^20^ and mice^19^ and with the assumption that a local spreading or generalization of TeNT effects, for example, via blood circulation, is dose dependent.^33^, ^34^, ^35^ The absence of generalized TeNT effects as well as of an unwanted spreading to other muscle areas supports the safety of intramuscular TeNT when applied in low dose. A similar relation of toxic and beneficial effects is already known from botulinum neurotoxin, the safety of which has already proven itself in clinical use for over several decades.^32^, ^36^ We assume that, comparable with botulinum neurotoxin,^37^ TeNT does not evoke undesired generalized effects as long as a certain dose is not exceeded.

Another potential obstacle to clinical use in humans is the fact that most people are vaccinated against TeNT. However, at least in immunized mice, TeNT effects could be reproduced despite a detectable titre level.^18^ It was speculated that the probability of circulating TeNT antibodies to bind TeNT may not be very high as compared with the exceptional high affinity of TeNT to neuronal membranes.^38^, ^39^, ^40^ Future studies are necessary on behalf of the immunization issue.

A limitation of our current study is that follow‐up assessment was only performed at a single time point. This was chosen because of logistic considerations as dog owners had to accept longer journeys to participate. So, our study provides limited data on the duration of TeNT effects in dogs. At least previous mice studies found the effects of TeNT to last similar durations as that of botulinum neurotoxin.^19^, ^41^, ^42^ But we do not know whether this similarity holds true for TeNT injections in dogs or yet in humans.

The results of this randomized controlled study demonstrate for the first time that intramuscular injections of TeNT are capable of focally reversing SCI‐related muscle atrophy. In addition, TeNT injections are well tolerated and seem to be safe, when administered at low dose. This beneficial effect on muscle atrophy alone may be associated with secondary therapeutic implications. A reverse of muscle atrophy in SCI patients could, for example, reduce the risk of secondary diseases such as pressure sores or bone fractures as these are mainly caused by immobility, reduced muscular activity, and disuse.^7^ An effective prophylaxis of which is urgently needed, as these sequels of SCI are clearly connected with reduced activities of daily living and thus a reduced quality of life.^1^ But above its application in paraplegia, also other neurological diseases, which are characterized by an impairment of the upper motor neuron, may be worthwhile to be investigated for potential therapeutic TeNT effects, for example, such as stroke or multiple sclerosis.

## Funding

The study was supported by a grant to A.K. of the Faculty of Medicine, Georg‐August‐Universität Göttingen (research programme).

## Conflict of interest

None declared.
